# Opiate-Induced Suppression of Rat Hypoglossal Motoneuron Activity and Its Reversal by Ampakine Therapy

**DOI:** 10.1371/journal.pone.0008766

**Published:** 2010-01-19

**Authors:** Amanda R. Lorier, Gregory D. Funk, John J. Greer

**Affiliations:** Division of Neuroscience, University of Alberta, Edmonton, Canada; Emory University, United States of America

## Abstract

**Background:**

Hypoglossal (XII) motoneurons innervate tongue muscles and are vital for maintaining upper-airway patency during inspiration. Depression of XII nerve activity by opioid analgesics is a significant clinical problem, but underlying mechanisms are poorly understood. Currently there are no suitable pharmacological approaches to counter opiate-induced suppression of XII nerve activity while maintaining analgesia. Ampakines accentuate α-amino-3-hydroxyl-5-methyl-4-isoxazole-propionate (AMPA) receptor responses. The AMPA family of glutamate receptors mediate excitatory transmission to XII motoneurons. Therefore the objectives were to determine whether the depressant actions of μ-opioid receptor activation on inspiratory activity includes a direct inhibitory action at the inspiratory premotoneuron to XII motoneuron synapse, and to identify underlying mechanism(s). We then examined whether ampakines counteract opioid-induced depression of XII motoneuron activity.

**Methodology/Principal Findings:**

A medullary slice preparation from neonatal rat that produces inspiratory-related output in vitro was used. Measurements of inspiratory burst amplitude and frequency were made from XII nerve roots. Whole-cell patch recordings from XII motoneurons were used to measure membrane currents and synaptic events. Application of the μ-opioid receptor agonist, DAMGO, to the XII nucleus depressed the output of inspiratory XII motoneurons via presynaptic inhibition of excitatory glutamatergic transmission. Ampakines (CX614 and CX717) alleviated DAMGO-induced depression of XII MN activity through postsynaptic actions on XII motoneurons.

**Conclusions/Significance:**

The inspiratory-depressant actions of opioid analgesics include presynaptic inhibition of XII motoneuron output. Ampakines counteract μ-opioid receptor-mediated depression of XII motoneuron inspiratory activity. These results suggest that ampakines may be beneficial in countering opiate-induced suppression of XII motoneuron activity and resultant impairment of airway patency.

## Introduction

Respiratory depression induced by opioid analgesics is a significant clinical problem [1]. This includes opiate-induced depression of hypoglossal motoneuron (XII MN) output, which activates the genioglossal muscle of the tongue and helps maintain an open airspace for effective breathing [2], [3]. Children and adults with obstructive sleep apnea (OSA) are particularly prone to opioid-induced apnea [4], [5], [6], [7], [8]. The mechanisms underlying opioid-induced suppression of XII nerve activity [9] are not well understood. Further, the current therapy to overcome the respiratory depression of administering μ-opiate receptor antagonists, such as naloxone, has the distinctly undesirable effect of removing analgesia. However, recent work [10], [11] has demonstrated that positive modulators of the AMPA sub-type of glutamate receptor, AMPAKINES, alleviate the opiate-induced depression of central respiratory rhythmogenesis [9], which is hypothesized to emanate from the preBötzinger complex (preBötC), without affecting analgesia. These data have led us to first assess whether an opiate-mediated inhibition of XII MNs contributes to the opiate-induced depression of breathing, and second, to examine the utility of AMPAKINES in reversing the opiate-mediated inhibition of XII inspiratory motor output. AMPAKINES are a family of compounds that modulate the actions of the excitatory neurotransmitter glutamate at AMPA receptors by altering channel kinetics [12], [13]. AMPA-receptor mediated currents are important for transmitting inspiratory drive to XII MNs [14], [15]. Thus, we hypothesized that increasing the charge transfer of inspiratory drive currents with AMPAKINES would counter μ-opiate receptor-mediated suppression of XII MN excitability. In this study we used medullary slice preparations from neonatal rats that produce inspiratory-related output in vitro to: i) determine the action of μ-opioid receptor activation on XII MN inspiratory activity; ii) determine the mechanism and sites of actions of μ-opioids within the XII motor nucleus, and iii) test the hypothesis that AMPAKINES counter opioid-induced depression of XII MN activity.

We examined the effects of two structurally distinct AMPAKINES, CX717 and CX614, which are classified as low- and high-impact AMPAKINES, respectively, based on differences in binding site, and the degree to which they modulate desensitization, deactivation and channel open probability [16], [17].

## Methods

### Medullary Slice and Extracellular Nerve Recordings

All experimental procedures were approved by University of Alberta Faculty of Medicine Animal Welfare Committee (Edmonton, AB). To examine the effects of [D-Ala(2),N-Me-Phe(4),Glyol(5)]-enkephalin (DAMGO) and AMPAKINE compounds on XII MN activity, rhythmically-active medullary slices obtained from neonatal (postnatal day 0 - 4, P0 - P4) Wistar rats were used. Animals were anaesthetized through inhalation of isoflurane and decerebrated. Rhythmic medullary slices were obtained by previously described methods [18]. Briefly, the brainstem-spinal cord was then isolated in cold, artificial cerebrospinal fluid (aCSF) containing (mM): 120 NaCl, 3 KCl, 1.0 CaCl_2_, 2.0 MgSO_4_, 26 NaHCO_3_, 1.25 NaH_2_PO_4_, 20 D-glucose, equilibrated with 95% O_2_/5% CO_2_. The brainstem-spinal cord was pinned to a wax chuck and serial 100–200 µm sections were cut in the rostral to caudal direction using a vibratome (Leica VT1000S, Nussloch, Germany). The 700 µm rhythmic slice extended from this rostral margin to the obex caudally and contained the preBötC, rostral ventral respiratory group, most of the XII motor nuclei and the rostral XII nerve rootlets. Slices were placed caudal surface up in a glass-bottomed recording chamber (500 µL) that was mounted on an upright microscope (Zeiss Axioscope II FS). Slices were held in place via nylon threads stretched over a platinum wire flattened into a horseshoe shape, and were continuously perfused with recirculating aCSF (flow rate ∼2 mL/min) aerated with 5% CO_2_/95% O_2_. At least 30 minutes prior to the start of data collection, the concentration of K^+^ in the aCSF was raised from 3 to 9 mM to ensure the production of long-term, stable inspiratory-related rhythm.

XII MN population activity was recorded from the medullary slice preparation using glass suction electrodes (A-M Systems, Carlsborg, WA) placed over the severed ends of XII nerve rootlets. Signals were amplified (5000X), band pass filtered (100 Hz–5 kHz), rectified, integrated (τ = 25 ms) and then saved to a computer via a Digidata 1322 A/D board (sampling rate = 2–5 kHz, Axon instruments).

### Whole-Cell Recording

A horizontal puller (Sutter P-97) was used to pull patch pipettes (3–4.5 MΩ) from filamented borosilicate glass (Clark/WPI, 1.2 mm outside diameter). Patch pipettes were filled with intracellular solution (ICS) containing (in mM): 1) 140 potassium-gluconate, 5 KCl, 5 NaCl, 1 CaCl_2_, 1 MgCl_2_, 10 HEPES, 10 EGTA, and 1 glucose. The pH of the ICS was adjusted to 7.2–7.3 using KOH.

The XII nucleus was clearly discernable in the slice preparation and whole cell recordings were obtained from inspiratory XII MNs located within ∼70 µm of the slice surface. Recordings were established under direct visualization using a CCD camera (ICD-47; Ikegami, Tokyo, Japan) and monitor attached to a Zeiss Axioscope II FS upright microscope equipped for infra-red differential interference contrast microscopy [19]. Whole-cell signals (membrane current or voltage) were amplified and filtered with a patch-clamp amplifier (2–5 kHz low-pass filter, Multiclamp 700A, Axon Instruments) and acquired with a 1322 A/D board and pClamp 9.0 software. Signals were displayed on a computer monitor and stored to computer hard disc. Off-line analysis of whole-cell signals was performed using Clampfit software. Series resistance (R_s_) and whole-cell capacitance (C_m_) were estimated as done previously [20], [21] using the R_s_ and C_m_ compensation features of MultiClamp Commander software to manually correct the current response to square-wave voltage pulses (100 Hz,−10 mV, 3 ms) under voltage-clamp conditions.

### Drugs and Their Application

The following drugs were used: DAMGO, tetrodotoxin (TTX), strychnine, bicuculline, (all from Tocris, Ellsville, MO), alpha-amino-3-hydroxy-5-methyl-4-isoxazolepropionic acid (AMPA), and glutamate (both from Sigma, St Louis, MO). The AMPAKINE compounds, CX614 and CX717, were provided by Cortex Inc (Dr. Mark Varney CEO, Irvine CA). Stock solutions of AMPAKINES were made in DMSO (final concentration of DMSO ≤0.02% (bath application) or ≤0.4% (local application)) and frozen in aliquots. DMSO alone at these concentrations is without effect on baseline inspiratory behaviour [21], [22]. Stock solutions of all other drugs were made in standard aCSF and frozen in aliquots. The final concentration of K^+^ in the drug solutions was matched to that of the aCSF.

Drugs were either applied to the bath or locally applied with microinjection from triple-barreled pipettes (tip diameter ∼5–6 µm per barrel O.D.) pulled from borosilicate glass capillaries (World Precision Instruments, Sarasota, FL). For local microinjection, drug pipettes were placed under microscope visualization so that the tip was just above the surface of the slice in the same relative position for each neuron. Drug microinjections (∼10 psi) were controlled via a programmable stimulator (Master-8, AMPI Instruments, Jerusalem, Israel) linked to a solenoid.

### Data Analyses

In most cases data are reported as the mean value relative to control ± S. E. of the mean. For data sets that were normally distributed (Kolmogorov-Smirnov test, GraphPad Prism 4.0), differences between means were identified using the Student t-test when only two groups were compared while a one-way ANOVA with the Tukey Multiple Comparison post test was used (GraphPad Prism 4.0) when more than two groups were compared. The non-parametric Wilcoxon matched pairs test (for two data groups) or Friedman test with Dunns post test (for greater than two groups) (GraphPad Prism 4.0) were used to assess differences between data sets that were not normally distributed. Values of p<0.05 were assumed significant.

The effect of bath application of drugs on XII nerve inspiratory output was assessed using Clampfit software to measure the resulting average change in XII inspiratory burst amplitude, area, duration and frequency during a two minute period 15 minutes after the drug was applied to the bath. Baseline and threshold levels for bursts were set using Clampfit software. Bursts were then automatically detected so that amplitude, area, duration and frequency were measured. The area of the burst was calculated for the region of the burst that was above the baseline. The values for this period were then compared to the average values over the two minute control period prior to drug application. Washout data were obtained using the same method 30 minutes after the drug washout began.

The effect on XII nerve inspiratory activity of locally applying drugs was assessed by measuring the resulting change in XII inspiratory burst amplitude, area, duration and frequency during the two minutes following drug application. The average values for this period were then compared to the average values over the two minute control period prior to drug application. Washout data were obtained by measuring average values over a two minute period occurring 15 minutes after the termination of drug application.

Drug-induced currents were measured by calculating the difference between the control or baseline (measured between inspiratory bursts) membrane current measured two minutes prior to drug application, and the peak membrane current during the two minute period following drug application. Drug-induced changes in the input resistance (R_N_) of inspiratory XII MNs were calculated based on current responses to voltage ramps (1.5 s duration, from −90 to −40 mV) applied before, during and after application of drug. Input resistance was estimated from the inverse slope of the linear portion of the current-voltage (I/V) relationship. The voltage-dependence and reversal potential of drug-induced currents were calculated by subtracting control I/V relationship from the I/V relationship obtained during the drug application. The effect of drug on inspiratory synaptic currents in XII MNs was also examined. The average amplitude and area of inspiratory synaptic currents during the two minutes following drug application were compared to those obtained for the control period prior to drug application.

To examine whether a drug acted via pre- or postsynaptic mechanisms to modulate the inspiratory output from XII MNs, the effect of the drug on miniature excitatory postsynaptic currents (EPSCs) that occur in the presence of tetrodotoxin (TTX, 1 µM), strychnine (10 µM) and bicuculline (10 µM) (all bath applied) was assessed. Membrane current was first recorded (5 kHz, Clampex) for 10–15 minutes in control conditions. Drugs were then applied to the bath and following a 15 minute “wash in” period membrane current was recorded again for a minimum of 10 min during the application of the drug of interest. Washout data was obtained similarly for a ∼10 minute period occurring at least 30 minutes after initiation of drug washout. On average we sampled ∼500 EPSCs during control and drug for each cell. Series resistance was closely monitored throughout these recordings and the MN was rejected if there was any change between control and drug conditions. In each condition the frequency and amplitude of EPSCs was measured using a custom designed template search protocol (Clampfit software). The effect of the drug on these parameters was determined by comparing cumulative frequency histograms for changes in the average mEPSC frequency and amplitude values between periods of control and drug application.

## Results

### Section A: Effects of DAMGO on XII Inspiratory Activity

#### A1) Bath application of DAMGO depresses inspiratory activity in the medullary slice preparation

**Figure 1:**
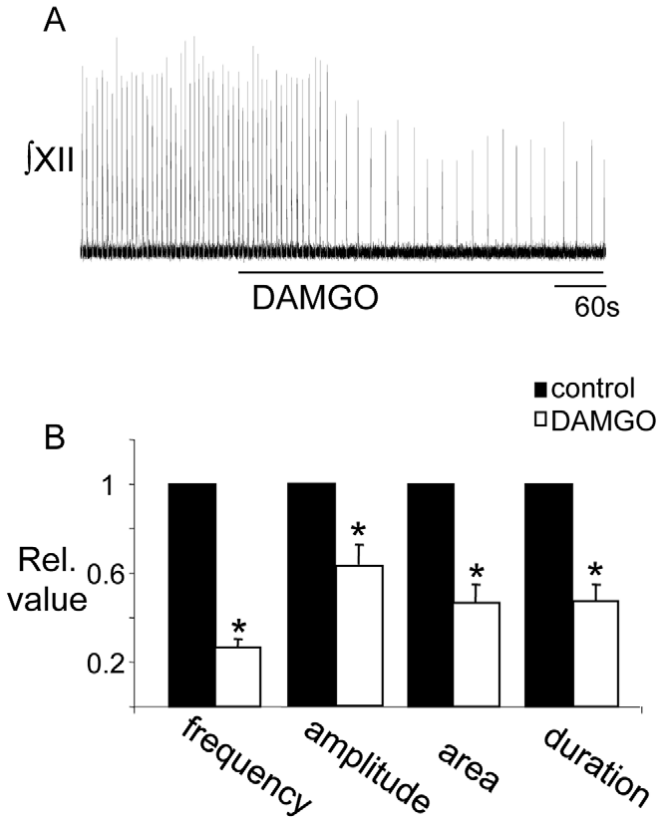
Bath application of DAMGO depresses inspiratory activity. A) Integrated (∫) XII nerve activity recorded from a medullary slice preparation illustrating that bath application of DAMGO (500 nM) depresses inspiratory activity. B) Group data (n = 12) showing the effect of bath-applied DAMGO (500 nM) on XII inspiratory burst frequency amplitude, area and duration relative to control. *  =  significantly different from control (p<0.05).

Application of the µ-opioid receptor agonist, DAMGO (500 nM, bath), to the in vitro rhythmically-active medullary slice preparation caused a profound depression of respiratory activity (Fig 1A). Recordings from XII nerve rootlets showed that subsequent to the addition of DAMGO, inspiratory burst frequency, amplitude, area and duration decreased to 0.26±0.04, 0.63±0.9, 0.46±0.08 and 0.47±0.07 of control respectively (n = 23, p<0.05, Fig 1B).

#### A2) Local application of DAMGO to the XII nucleus reduces inspiratory burst amplitude

**Figure 2:**
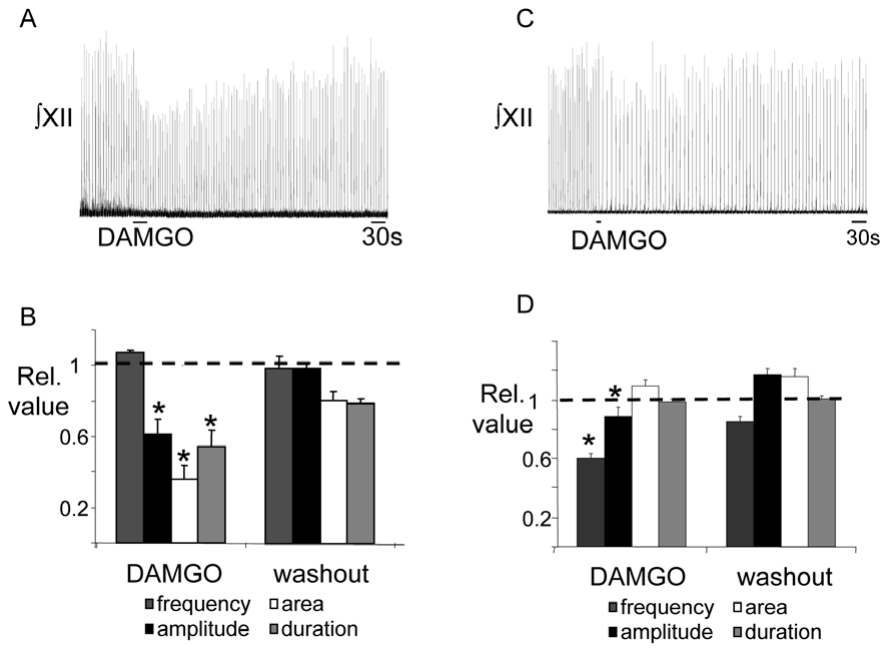
Local application of DAMGO to the XII nucleus depresses inspiratory burst amplitude, area and duration. A) ∫XII nerve activity recorded from a medullary slice preparation demonstrating that local application of DAMGO (5 µM, 30s) into the XII nucleus decreases inspiratory burst amplitude. B) Group data (n = 5) show that local application of DAMGO (5 µM, 30 s) significantly reduces XII inspiratory burst amplitude, area and duration. C) ∫XII nerve activity recorded from a medullary slice preparation demonstrating that local application of DAMGO (5 µM, 10s) into the preBötC decreases inspiratory burst amplitude and frequency. D) Group data (n = 10) show that local application of DAMGO (5 µM, 10 s) significantly reduces XII inspiratory burst amplitude and frequency. *  =  significantly different from control (p<0.05).

To establish whether μ-opioid receptor activation has a direct affect on inspiratory burst amplitude, we locally applied DAMGO to the XII motor nucleus. Local application of DAMGO (5 µM, 30s; n = 5) to the XII nucleus of medullary slice preparations significantly decreased inspiratory motor output recorded from the XII nerve (Fig 2A and B). The amplitude of inspiratory bursts decreased to 0.61±0.08 of control (p<0.001), inspiratory burst area decreased to 0.36±0.08 of control (p<0.01), and inspiratory burst duration decreased to 0.54±0.09 (p<0.01) of control (Fig 2B). These inhibitory actions on burst amplitude gradually reversed with washout of DAMGO. There was no change in inspiratory burst frequency (1.07±0.01 of control) following local DAMGO application in the XII nucleus. Interestingly, local application of DAMGO (5 µM, 10s; n = 10) into the preBötC also reduced both inspiratory XII burst amplitude and frequency to 0.89±0.06 (p<0.05) and 0.60±0.03 (p<0.001) of control respectively (Fig 2C and D). This is consistent with the localization of XII preMNs adjacent (dorsal) to the preBötC region [23].

**Figure 3:**
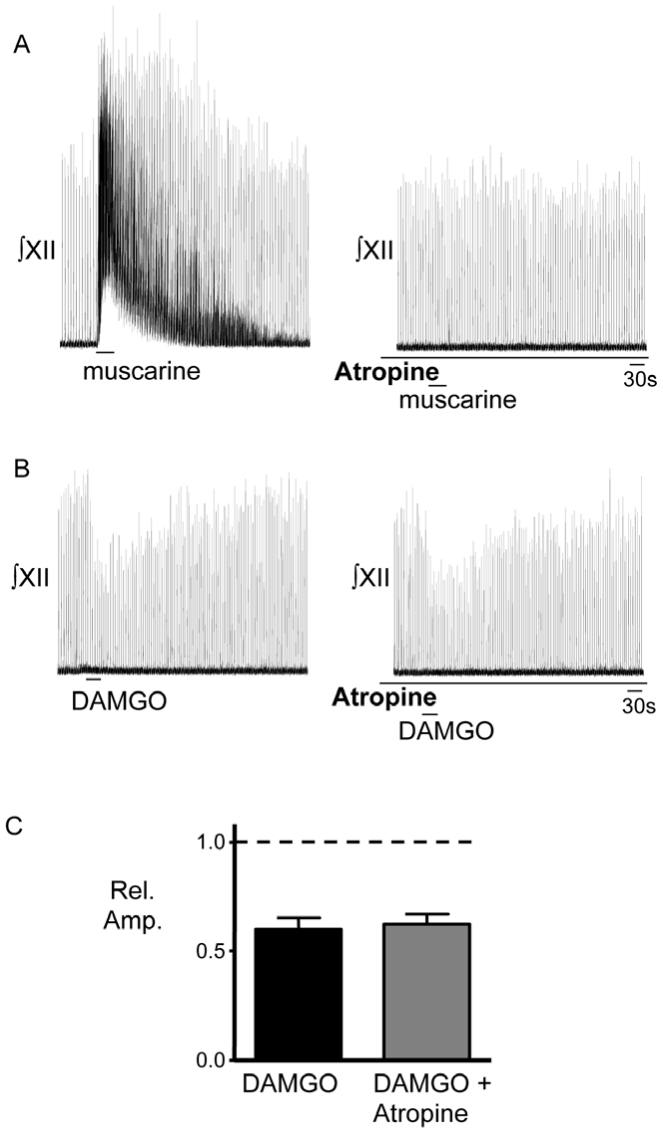
Muscarinic receptors do not contribute to the DAMGO-evoked depression of XII inspiratory activity. A) ∫XII nerve activity recorded from a medullary slice preparation illustrating that local application of muscarine (100 µM, 30 s) into the XII motor nucleus (top trace) evokes tonic discharge and increases inspiratory burst amplitude and that this response is inhibited by atropine (bath, 1 µM, bottom trace.) B) ∫XII nerve activity recorded from the same slice preparation as in (A) showing that the inhibition of XII inspiratory burst amplitude evoked by local application of DAMGO (5 µM, 30 s) in the XII nucleus (top and bottom traces) is not reduced by bath-applied atropine (1 µM, bottom trace). C) Group data (n = 4) showing that the magnitude of DAMGO-mediated inhibition of XII inspiratory burst amplitude is not affected by bath-applied atropine.

Data from rat in vivo studies demonstrate that opiates can depress XII MN activity in part via an increase in acetylcholine release [24]. Thus, to assess whether the DAMGO-induced depression of XII MN inspiratory activity was due to the activation of muscarinic acetylcholine receptors, we locally-applied DAMGO (5 µM, 30 s; n = 4) to the XII nucleus before, during and after bath application of the general muscarinic receptor antagonist, atropine (1 µM). Atropine, at concentrations that blocked the response to locally-applied muscarine (100 µM, 30 s, n = 4) (Fig 3A), failed to block the DAMGO-evoked reduction in XII nerve inspiratory burst amplitude (Fig 3B). Burst amplitude decreased to 0.60±0.06 and 0.62±0.05 of control in DAMGO and atropine respectively (Fig 3C, n = 4).

#### A3) DAMGO reduces inspiratory synaptic current amplitude but does not change the input resistance of XII MNs

**Figure 4:**
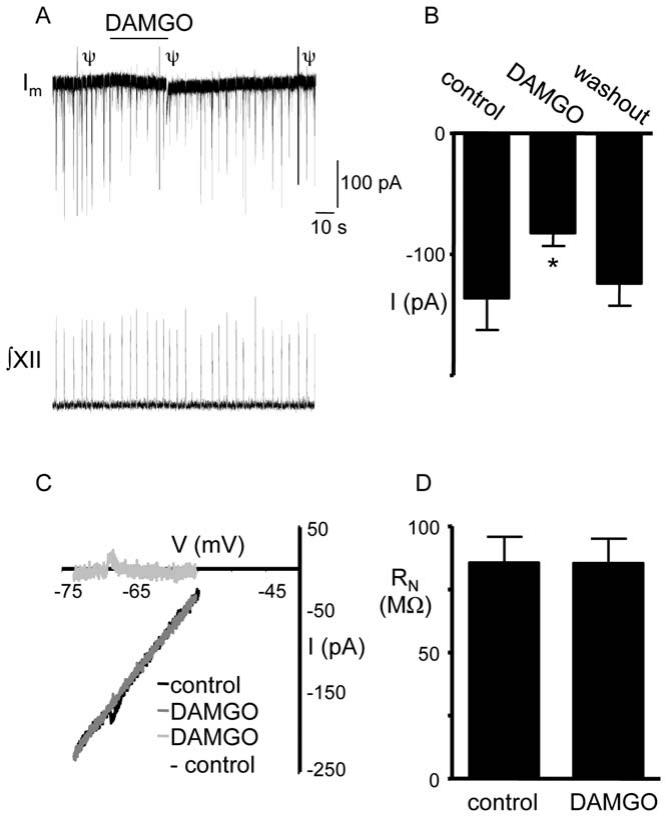
Local application of DAMGO to XII MNs reduces inspiratory synaptic current amplitude. A) ∫XII nerve activity and voltage-clamp (V_hold_ = −60 mV) recording of membrane current (I_m_) from an inspiratory XII MN illustrating the response to locally-applied DAMGO (5 µM, 30 s). B) Group data (n = 9) show that locally-applied DAMGO (5 µM, 30 s) significantly reduces the amplitude of inspiratory synaptic currents. C) Whole-cell I/V relationships obtained before (control) and during application of DAMGO application to an inspiratory XII MN. Subtraction of the control I/V from that obtained in DAMGO produces the I/V relationship for the DAMGO current (DAMGO – control). D) Group data (n = 9) showing that DAMGO had no effect on the R_N_ of inspiratory XII MNs. * = significantly different from control (p<0.05). ψ = transients resulting from delivery of the I/V ramp.

Having established that DAMGO depresses the inspiratory motor output recorded from the XII nerve, the mechanism underlying this effect was further examined with whole-cell recordings. Only inspiratory XII MNs (i.e. those that showed distinct, inward inspiratory synaptic currents in phase with inspiratory bursts in the XII nerve) were examined in this study. DAMGO (5 µM, 30 s) was locally applied to 9 inspiratory MNs with an average R_N_ of 86±15 MΩ that received inspiratory synaptic currents averaging -136±35 pA. DAMGO significantly reduced inspiratory synaptic current amplitude from -136±35 pA to -82±15 pA (0.67±0.05 of control, p<0.01; n = 9) (Fig 4A and B) and inspiratory burst area to 0.56±0.04 of control (p<0.001: n = 9). Despite these effects on synaptic currents, DAMGO had no effect on membrane current in XII inspiratory MNs at a holding potential of −60 mV, or between −75 and −55 mV, as shown for a single MN (Fig 4A and C). The average baseline current at a holding potential of −60 mV was −163±33 pA during control and −164±34 pA following DAMGO application (n = 9). DAMGO was also without effect on the R_N_ of inspiratory XII MNs. R_N_ averaged 86±15 MΩ in control and 85±14 MΩ following DAMGO application (n = 9) (Fig 4C and D).

#### A4) DAMGO reduces the frequency of miniature EPSCs recorded from XII inspiratory MNs

**Figure 5:**
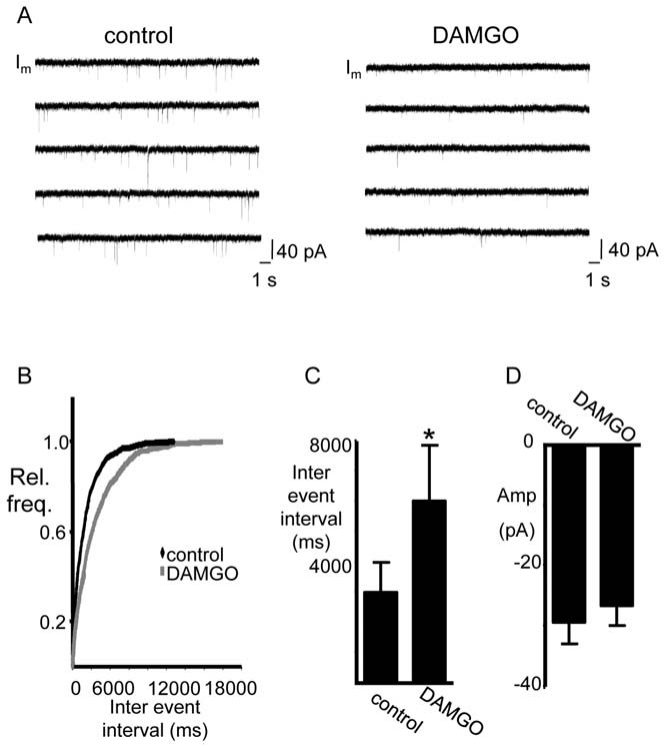
Bath application of DAMGO reduces the frequency of mEPSCs in inspiratory XII MNs. A) Voltage-clamp (V_hold_ = −60 mV) recording of membrane current (I_m_) from an inspiratory XII MN in the presence of TTX (1 µM), strychnine (10 µM) and bicuculline (10 µM) before (right trace) and after (left trace) bath application of DAMGO (500 nM). B) Cumulative frequency histogram for one XII MN showing that DAMGO increased the inter-event interval of mEPSCs. C and D) Group data (n = 6) illustrate that DAMGO evokes a significant increase in the mEPSC inter-event interval but not the mEPSC amplitude. *  =  significantly different from control (p<0.05).

Given that DAMGO had no noticeable effect on the membrane properties of XII MNs, we next assessed whether the DAMGO-evoked reduction in inspiratory burst amplitude reflected an action on glutamatergic synaptic transmission at the pre-motor to XII MN synapse. The effect of DAMGO on the frequency and amplitude of mini EPSCs recorded from inspiratory XII MNs was assessed with whole-cell recordings made in the presence of bath-applied TTX (1 µM), strychnine (10 µM), and bicuculline (10 µM) to block action potentials and GABA and glycinergic IPSCs, respectively. DAMGO (500 nM, bath) significantly decreased the frequency but not the amplitude of mini EPSCs (Fig 5A-D). The average period (or inter event interval) of mini EPSCs increased from 3007±1081 ms to 6021±2019 ms (n = 6, p<0.05) (Fig 5C). In contrast, the amplitude of mini EPSCs was −29±4 pA in control and −27±4 pA in DAMGO (n = 6, Fig 5D). Washout data were obtained for three of the six neurons. In this group of three MNs the average period increased from 2781±747 ms to 6354±1454 ms in DAMGO (p<0.01) and then recovered partially to 4087±1557 ms following washout of DAMGO (data not shown).

### SECTION B: Effects of Ampakines on DAMGO-Induced Depression of XII Inspiratory Activity

#### B1) The AMPAKINES CX614 and CX717 alleviate opioid-induced inspiratory depression

**Figure 6:**
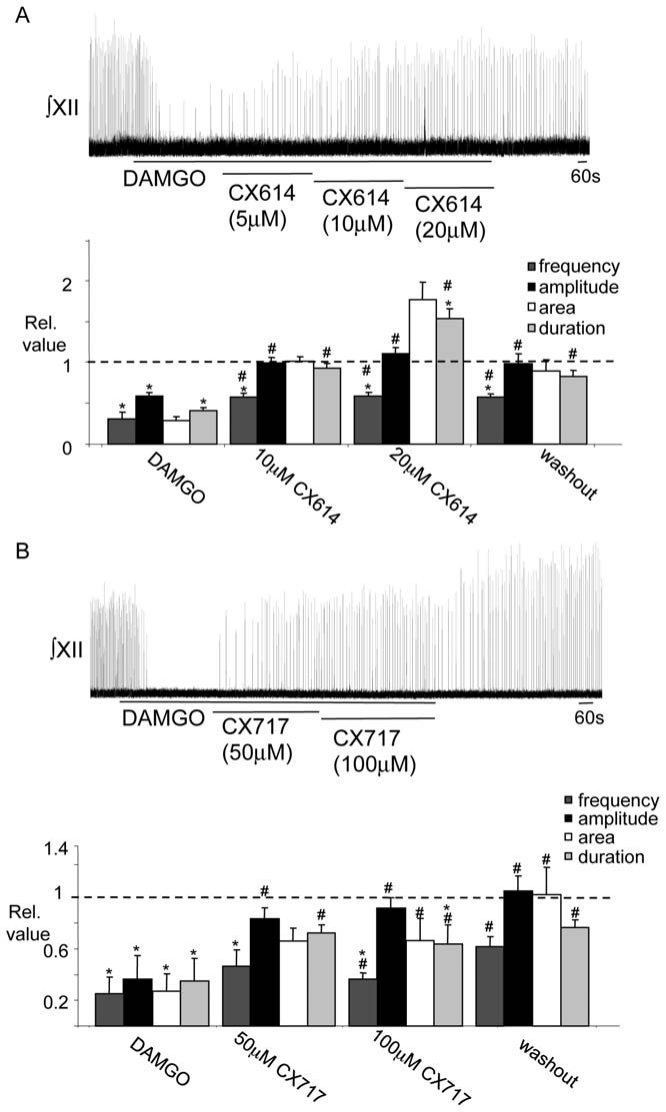
Bath application of CX614 or CX717 counters DAMGO-induced inspiratory depression. ∫XII nerve activity recorded from a medullary slice preparation and group data illustrating that the depression of respiratory activity evoked by bath-applied DAMGO (500 nM) was significantly attenuated by bath application of (A) CX614 (5–20 µM, n = 5) or (B) CX717 (50–100 µM, n = 6). Expanded time scale excerpts from control, DAMGO and AMPAKINE conditions are shown on the right hand side of the continuous time trace in (A) and (B). *  =  significantly different from control, #  =  significantly different from DAMGO (p<0.05).

Bath application of DAMGO (500 nM) profoundly depresses inspiratory activity recorded from the XII nerve (see A1 above). This depression of XII nerve activity was significantly alleviated by bath application of either CX614 or CX717 (Fig 6). In the CX614 (10–20 µM) experiments (n = 5), DAMGO reduced inspiratory burst frequency, amplitude, area and duration to 0.33±0.07, 0.58±0.05, 0.29±0.05, and 0.41±0.04 of control, respectively. At 10 µM CX614, inspiratory burst frequency, amplitude, area and duration recovered to 0.59±0.04, 0.99±0.06, 1.01±0.05 and 0.93±0.05 of control respectively (Fig 6). Following an increase of CX614 to 20 µM the values for inspiratory burst frequency, amplitude, area and duration were 0.59±0.04, 1.11±0.08, 1.76±0.22 and 1.53±0.12 (Fig 6) of control, respectively. These reflect significant 2.24±0.71, 1.93±0.15, 6.49±0.80 and 3.76±0.25-fold increases from the respective DAMGO values (Fig 6, p<0.05).

In the CX717 experiments, DAMGO reduced inspiratory burst frequency, amplitude, area and duration to 0.25±0.12, 0.36±0.18, 0.27±0.14 and 0.35±0.17 of control, respectively (n = 6). With the subsequent addition of CX717 (50 µM) to the bath, inspiratory burst amplitude and duration recovered to 0.83±0.8, and 0.72±0.06 relative to control respectively. These represented increases of 1.19±0.05 and 1.18±0.06 relative to DAMGO values, respectively (p<0.05). Inspiratory frequency and burst area increased numerically (frequency: 0.25±0.12 (DAMGO) to 0.46±0.13 (CX717), area: 0.27±0.14 (DAMGO) to 0.66±0.1 (CX717), but these were not statistically significant. When CX717 was applied at 100 µM, however, significant recovery was observed in all parameters. Inspiratory frequency, amplitude, area and duration increased to; 0.36±0.05, 0.92±0.08, 0.66±0.17 and 0.63±0.15 of control, respectively (Fig 6, p<0.05).

#### B2) Local application of CX614 and CX717 to the XII nucleus increases inspiratory burst amplitude following DAMGO-induced depression

**Figure 7:**
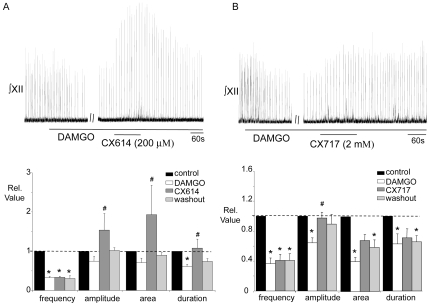
Local application of CX614 or CX717 to the XII nucleus counters DAMGO-induced depression of XII inspiratory burst amplitude. ∫XII nerve activity from a medullary slice preparation and group data illustrating that the depression of inspiratory burst amplitude, duration and area evoked by bath application of DAMGO (500 nM) was significantly reduced by local application of (A) CX614 (200 µM, n = 6) or (B) CX717 (2 mM, n = 5) into the XII nucleus. #  =  significantly different from DAMGO (p<0.05).

Since bath application of AMPAKINES following DAMGO depression increased inspiratory burst amplitude as well as frequency, we hypothesized that these compounds had a direct effect in the XII nucleus to modulate amplitude. To test this we locally injected CX614 or CX717 to the XII nucleus subsequent to depressing inspiratory output by bath applying DAMGO (500 nM). In this series of experiments bath application of DAMGO significantly depressed inspiratory burst amplitude frequency, area and duration recorded from the XII nerve to 0.72±0.1, 0.31±0.05, 0.5±0.09 and 0.6±0.08 relative to control, respectively (n = 11). Subsequently, local application of CX614 (200 µM, 60–120s) to the XII nucleus in six of these experiments significantly increased inspiratory burst amplitude, area and duration by 1.5±0.3, 2.6±0.8 and 1.5±0.4 fold relative to the level observed in DAMGO, respectively (n = 6: Fig 7A). There was no effect on inspiratory frequency. Similarly in the second group of experiments, local application of CX717 (2 mM, 120 s) to the XII nucleus evoked a significant increase in XII nerve inspiratory burst amplitude (by 1.5±0.1 fold relative to DAMGO: n = 5) without affecting inspiratory burst area, duration or frequency (Fig 7B). Local application of CX717 in control conditions (i.e. without prior initiation of an opioid-induced respiratory depression) had no effect on the amplitude of inspiratory bursts from the XII nerve (n = 3, not shown). However, local application of CX614 produced a small but significant increase in burst amplitude (1.12±0.04 fold greater than control, n = 4, data not shown).

#### B3) CX614 and CX717 increase XII MN inspiratory synaptic current amplitude following DAMGO-induced depression

**Figure 8:**
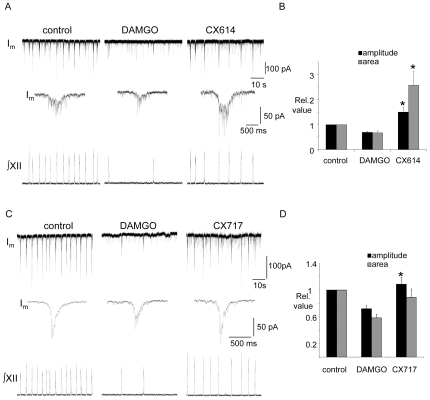
CX614 and CX717 potentiate XII MN inspiratory synaptic currents following a DAMGO-induced depression of inspiratory activity. ∫XII nerve activity and voltage-clamp (V_hold_ = −60 mV) recording of membrane current (I_m_) from an inspiratory XII MN illustrating the effects of bath-applying first DAMGO (500 nM) and then (A) CX614 (20 µM) or (C) CX717 (200 µM). Middle traces show an inspiratory synaptic current envelope averaged from five inspiratory cycles in each condition. Group data showing the effects of DAMGO and, in the continued presence of DAMGO, the effects of CX614 (B, n = 7) or CX717 (D, n = 8) on the amplitude and area of inspiratory synaptic currents. #  =  significantly different from DAMGO (p<0.05).

The effect of AMPAKINES on XII MN inspiratory synaptic currents was assessed using whole –cell recordings from inspiratory XII MNs. Either CX614 (20 µM) or CX717 (200 µM) was bath applied after inspiratory activity was depressed with DAMGO (500 nM, bath). Bath-applied DAMGO (n = 15) significantly reduced inspiratory synaptic current amplitude and area (charge transfer) to 0.7±0.03 and 0.6±0.04 of control, respectively (Fig 8A, B). Bath application of CX614 to seven of these neurons significantly increased both peak amplitude and charge transfer by 2.3±0.3 and 4±0.8 fold relative to DAMGO, respectively (Fig 8A, B, n = 7). Neither DAMGO nor CX614 altered R_N_ (1.11±0.03 and 1.06±0.06 relative to control, respectively; data not shown). Bath application of CX717 to the remaining eight neurons caused inspiratory synaptic current amplitude to significantly increase by 1.5±0.1 fold relative to DAMGO (p<0.05) (Fig 8C, D). Charge transfer did not significantly increase from DAMGO levels, however, the charge transfer value in CX717 was no longer significantly reduced compared to control (p<0.05) (Fig 8C, D). Neither DAMGO nor CX717 had a significant effect on the R_N_ of XII inspiratory MNs (1.07±0.02 and 1.01±0.04 relative to control, respectively; data not shown).

#### B4) Ampakines may act on XII inspiratory MNs through a postsynaptic mechanism

**Figure 9:**
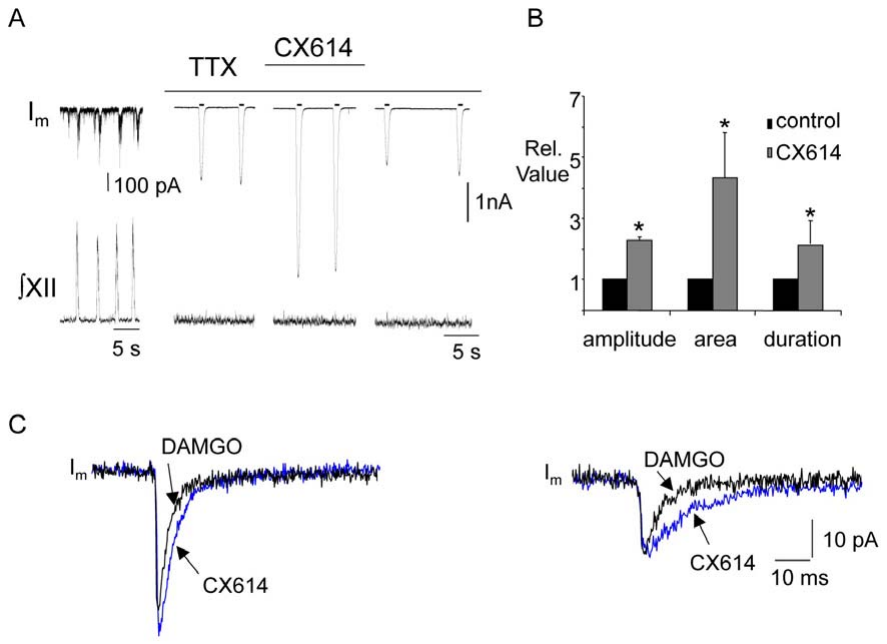
CX614 acts postsynaptically to potentiate AMPA or glutamate-evoked currents in XII MNs. A) Voltage-clamp (V_hold_ = −60 mV) recording of membrane current (I_m_) from an inspiratory XII MN before (left most trace, note different amplitude scale) and during bath application of TTX (1 µM). Brief pulses of AMPA or glutamate (indicated by short horizontal bar above current trace) were delivered before, during and after bath application of CX614 (20 µM). B) Group data (n = 5) showing the relative amplitude, area and duration of the AMPA/glutamate currents in control and during CX614. C) Voltage-clamp (V_hold_ = −60 mV) recording of membrane current (I_m_) from two different inspiratory XII MNs in the presence of TTX (1 µM), strychnine (10 µM), bicuculline (10 µM) and DAMGO (500 nM) illustrating the effect of CX614 (bath, 20 µM) on the shape of the mEPSCs. Each trace represents the average of 20–30 individual mEPSCs. *  =  significantly different from control (p<0.05).

Whole-cell recordings were used to examine the effect of CX614 on AMPA receptor currents in inspiratory XII MNs. Motoneurons were first identified as inspiratory based on inward synaptic currents in phase with XII bursts. Then, in the presence of TTX, we locally applied either glutamate (200 µM, 200 ms, n = 2) or AMPA (10 µM, 200 ms, n = 3) before, during and after bath application of CX614 (20 µM). Glutamate/AMPA receptor current amplitude, area and duration reversibly increased by 2.27±0.1, 4.34±1.5 and 2.12±0.75 fold, respectively, in the presence of CX614 (p<0.05, n = 5, Fig 9A, B).

To further investigate the mechanism underlying the CX614-mediated enhancement of inspiratory burst activity, we examined the effect of CX614 on mini EPSCs in XII inspiratory MNs. In the presence of TTX (1 µM), bicuculline (10 µM), strychnine (10 µM) and DAMGO (500 nM), bath application of CX614 (20 µM) had no effect on the inter-event interval (3853±1068 ms to 2375±498 ms) or amplitude (−37±6 pA to −35±5 pA) of mini EPSCs in inspiratory XII MNs (n = 5, data not shown). However, CX614 did significantly change the shape of the mini EPSCs by slowing decay kinetics (Fig 9C and Table 1).

**Table 1:** Effects of CX614 on mEPSC kinetics.

	*control (DAMGO)*	*CX614*
	Mean ± SEM	Mean ± SEM
**Time to Rise Half-amplitude (ms)**	0.75±0.02	0.81±0.03
**Time to Decay Half-amplitude (ms)**	6.32±0.11	6.66*±0.15
**Decay Tau (ms)**	3.37±0.14	3.9*±0.2
**Max Rise Slope (pA/ms)**	−42±7	−38±7
**Max Decay Slope (pA/ms)**	5.95±1.01	5.18±1.07
**Rise Slope 10% to 90% (pA/ms)**	−22.5±3.91	−19.6±4.08
**Decay Slope 90% to 10% (pA/ms)**	5.51±0.85	4.74±0.84
**Rise Time 10% to 90% (ms)**	1.37±0.02	1.43*±0,03
**Decay Time 90% to 10% (ms)**	5.070.3	5.24±0.12

* =  significantly different from control (DAMGO) (p<0.05).

**Figure 10:**
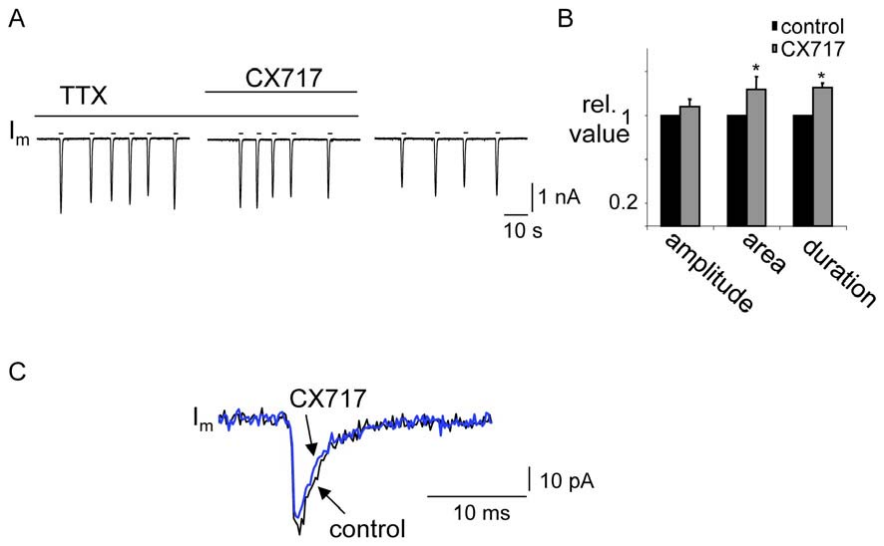
CX717 acts postsynaptically to potentiate AMPA or glutamate-evoked currents in XII MNs. A) Voltage-clamp (V_hold_ = −60 mV) recording of membrane current (I_m_) from an inspiratory XII MN during bath application of TTX (1 µM). Brief pulses of AMPA (indicated by short horizontal bar above current trace) were delivered before, during and after bath application of CX717 (200 µM). B) Group data (n = 5) showing the relative amplitude, area and duration of the AMPA currents in control and during CX717. C) Voltage-clamp (V_hold_ = −60 mV) recording of membrane current (I_m_) from an inspiratory XII MN in the presence of TTX (1 µM), strychnine (10 µM), bicuculline (10 µM) and DAMGO (500 nM) illustrating the shape of the mEPSC in control (DAMGO) and CX717 (bath, 200 µm). Each trace represents the average of 20–30 individual mEPSCs. *  =  significantly different from control (p<0.05).

We also examined the effect of CX717 on AMPA receptor currents in XII MNs. AMPA (10 µM, 400 ms, n = 5) was locally applied in the presence of TTX before, during and after bath application of CX717 (200 µM). Although AMPA receptor current amplitude was unaffected by CX717 (1.08±0.07 relative to control), current area (charge transfer) and duration increased by 1.23±0.1 and 1.25±0.04 fold (p<0.05, n = 5, Fig 10A, B).

The effect of CX717 on mini EPSCs in XII inspiratory MNs was also examined in the presence of TTX (1 µM), bicuculline (10 µM), strychnine (10 µM) and DAMGO (500 nM). Under these conditions, bath application of CX717 (200 µM) had no significant effect on either the inter-event interval (5296±3044 ms vs. 4396±1739 ms) or amplitude (−28±4 pA vs. −28±4 pA) of mini EPSCs (n = 5, data not shown). CX717 was also without effect on the shape of the mini EPSCs (Fig 10C and Table 2).

**Table 2:** Effects of CX717 on mEPSC kinetics.

	*control (DAMGO)*	*CX717*
	Mean ± SEM	Mean ± SEM
**Time to Rise Half-amplitude (ms)**	0.69±0.04	0.68±0.03
**Time to Decay Half-amplitude (ms)**	6.29±0.07	6.29±0.06
**Decay Tau (ms)**	3.20±0.04	3.20±±0.07
**Max Rise Slope (pA/ms)**	−31±5	−29±4
**Max Decay Slope (pA/ms)**	4.39±0.7	4.1±0.6
**Rise Slope 10% to 90% (pA/ms)**	−17±2.3	−16±2.2
**Decay Slope 90% to 10% (pA/ms)**	3.74±0.7	3.61±0.7
**Rise Time 10% to 90% (ms)**	1.36±0.02	1.35±0.01
**Decay Time 90% to 10% (ms)**	5.99±0.3	5.96±0.3

## Discussion

### μ-Opiate Receptor Effects on Inspiratory XII MN Activity

In this study we demonstrate that local application of DAMGO into the XII nucleus depresses the output of inspiratory XII MNs in neonatal preparations in vitro. These findings, which suggest that μ-opioid receptors are expressed within the XII nucleus and activation of these receptors modulates the activity of inspiratory XII MNs, are consistent with recent in vivo studies in adult rats [2]. However, the expression of μ-opioid receptors in the XII motor nucleus and also the mechanism underlying modulation of XII MNs by μ-opioids is controversial. μ-opioid receptor labeling in the XII nuclei of adult rat [25] and cat [26] is relatively sparse. In the adult cat none of the extremely few μ-opioid-receptor containing nerve processes found in the XII nucleus appear to make contact with genioglossus MNs [26]. In addition, no μ-opioid receptors were found on the soma. Thus, it was hypothesized that systemically administered morphine depresses genioglossus muscle activity [27] through mechanisms that operate outside the XII nucleus [26]. However, a different study found strong expression of μ-opioid receptors in the XII nucleus of embryonic, neonatal and adult rat [28]. Our data demonstrates both a direct action of μ-opioid-receptors within the XII MN pool and an indirect action via suppression within the region of the preBötC that contains XII pre-MN [23] .

In other parts of the CNS, the inhibitory actions of μ-opioid receptor activation are mediated via G protein-regulated mechanisms at pre and/or postsynaptic membranes. Presynaptically, μ-opioid receptor activation diminishes Ca^2+^-dependent,exocytotic neurotransmitter release by inhibiting voltage-dependent Ca^2+^channels, or by activating K^+^ channels, which raises the threshold and shortens the duration of action potentials [29], [30]. Postsynaptically, μ-opioid agonists activate at least two different K^+^ conductances, one of which is an inward rectifier [31]. Data from our study suggest that the DAMGO-induced depression of XII MN activity results from presynaptic modulation of glutamatergic transmission. This is supported by the observation that DAMGO, whether locally- or bath-applied, had no effect on XII MN membrane current or resistance, whereas it affects both parameters in μ-opioid receptor expressing rhythmogenic preBötC neurons [32]. In addition, DAMGO decreased the frequency, but not the amplitude, of miniature EPSCs.

The μ-opioid receptor-mediated depression of XII MN inspiratory output is hypothesized to involve ACh release and the subsequent presynaptic inhibition of glutamatergic transmission through activation of muscarinic receptors [24], however, this finding is controversial [2]. Our data in the neonatal rat medullary slice preparation suggest that activation of muscarinic receptors in the XII nucleus is excitatory. In addition, the DAMGO-mediated inhibition of XII inspiratory output in neonates in vitro was completely unaffected by blocking muscarinic receptors. While this discrepancy may reflect preparation differences (in vitro versus in vivo), it is more likely to reflect a developmental change in muscarinic modulation. As seen in our experiments, previous work in neonatal rats in vitro indicates that activation of mACh receptors on XII MNs causes depolarization, facilitates repetitive firing and potentiates XII inspiratory burst output [33], [34]. In contrast, activation of mACh receptors suppresses the activity of juvenile XII MNs in vitro, via presynaptic inhibition of evoked glutamatergic excitatory inputs [35]. This development change is consistent with the observation in adult rat that mAChR activation suppresses inspiratory-related activity of the genioglossus muscle [36].

### Effect of Ampakines

The high- (CX614) and low-impact (CX717) AMPAKINES both alleviated the DAMGO-induced depression of XII MN activity and inspiratory synaptic drive in a dose-dependent manner. Importantly, the effect of AMPAKINES was not restricted to the preBötC where the rhythmic drive is generated, because local application of AMPAKINES to the XII nucleus alone increased XII MN output following DAMGO-mediated depression. In addition, both AMPAKINES had postsynaptic actions on XII MNs. CX614 was particularly potent at increasing the amplitude, duration and area of postsynaptic currents induced by exogenous application of AMPA or glutamate. It also slowed the decay of mini EPSCs. These actions at inspiratory XII MN synapses are consistent with those cyclothiazide, which also inhibits AMPA receptor desensitization and slows deactivation [22] They are also consistent with those reported for AMPA/glutamate currents in hippocampal slices [37], and for the high-impact AMPAKINES in general which potently slow the kinetics of both channel closing and desensitization.

The effects of CX717 on XII MN responses were more subtle than those of CX614. CX717 increased the duration and area, but not the amplitude, of currents induced by exogenous AMPA/glutamate. The mechanisms by which CX717 and other low-impact AMPAKINES produce their actions are less well understood than for high impact AMPAKINES. They do not strongly modulate EPSC amplitude but rather cause slight changes in channel opening probability and open time [17], [38], [39], [40]. These changes can be difficult to detect in an EPSC averaged from many events at multiple unidentified synapses as done here, and this may contribute to the fact that the effect of CX717 on mini EPSCs did not reach statistical significance.

In addition to their direct effects on glutamatergic signaling, high impact AMPAKINES such as CX614 elevate the neuronal production of BDNF [41]. However, any actions via BDNF-mediated mechanisms are unlikely to play a role in the responses of XII MN measured in this study as the rapid kinetics of CX614 effects is inconsistent with an up-regulation of BDNF synthesis.

It is noteworthy that CX717 did not potentiate baseline XII MN nerve discharge and the only effect of CX614 on baseline activity was a small but significant increase in burst amplitude. Therefore, stimulatory effects of AMPAKINES were considerably more apparent following DAMGO-induced depression of XII MN activity. This is consistent with observations in vitro and in vivo that AMPAKINES only increase respiratory frequency after it is suppressed by opioid administration [11], [42]. Similarly, CX717 does not elevate respiratory or cardiovascular parameters under baseline conditions in human studies [43], [44], [45]. The mechanism underlying the preferential potentiation of suppressed synapses or networks by AMPAKINES is not fully understood [16], [46], but of obvious significance from a therapeutic perspective in that it will reduce the likelihood of side-effects. This is especially true for CX717 (and possibly all low-impact AMPAKINES), in that not only are its actions limited to depressed glutamatergic synapses, its potentiation of the depressed activity never exceeded baseline values (i.e. those that preceded DAMGO). CX614 similarly acted only on suppressed synapses. However, this high impact AMPAKINE elevated the burst envelope and synaptic currents to levels that exceeded baseline.

In summary, these data demonstrate that the marked suppression of XII MN inspiratory discharge by μ-opiate receptor agonists, which is primarily attributed to their actions at the level of the preBötC or premotor networks [47], is also due to direct actions at the glutamatergic, premotor-to-XII MN inspiratory synapse. More specifically, μ-opiate receptor agonists inhibit glutamate release from presynaptic terminals onto XII MNs. Data also demonstrate that AMPAKINES alleviate the opiate-induced depression of breathing and that this occurs in part through the modulation of AMPA receptors at XII MN synapses. Importantly, low-impact AMPAKINES are metabolically stable, do not cause unwanted side-effects and do not interfere with analgesia [11]. Thus, they hold promise as a potential pharmacological therapy to treat opiate-induced suppression of XII MN activity and the resultant loss of airway patency. It will be interesting to explore the utility of AMPAKINES in counteracting the depression of XII MN activity that contributes to other pathological conditions such as apnea of prematurity, obstructive sleep apnea and opioid-induced central apnea.
